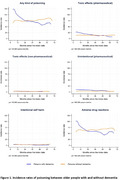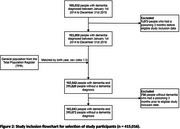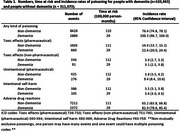# Incidence and profiles of poisonings in older people with dementia in Sweden: a matched registry‐based cohort study

**DOI:** 10.1002/alz70861_108531

**Published:** 2025-12-23

**Authors:** Ilsa R Wojt

**Affiliations:** ^1^ University of Sydney, Sydney, NSW Australia

## Abstract

**Background:**

Poisonings are a significant global public health challenge, especially for older adults with dementia. However, few studies have examined poisonings in this vulnerable group. This study aims to explore the incidence, types, and substances involved in poisonings among older people with and without dementia in Sweden.

**Method:**

This matched cohort study utilized linked Swedish national registries to examine poisonings. Participants aged 65 and over, with an initial dementia diagnosis between 2014 and 2018 were matched to controls by age and sex in a 1:3 ratio and followed until December 31, 2019. Poisoning cases were identified using ICD‐10 codes, and incidence rates (IR) and hazard ratios (HR) with 95% confidence intervals (CI) for poisonings were calculated using Kaplan‐Meier and survival regression models.

**Result:**

Overall, 415,016 people (103,943 with dementia and 311,073 without dementia) were included. The incidence of any kind of poisoning was higher in people with dementia than those without dementia (IR: 100.3 per 100,000 person‐months, 95%CI, 96.7–104.0 vs 76.4 per 100,000 person‐months, 95%CI 74.8‐78.1). Dementia was associated with a higher risk of any poisoning (HR: 1.48, 95%CI 1.39‐1.58), particularly from unintentional poisonings (HR: 2.48, 95%CI 1.96‐3.13) and adverse drug reactions (HR: 1.46, 95%CI 1.37‐1.57) in the six months following diagnosis. Psychotropics, anti‐thrombotics and cardiovascular medications were most associated with poisonings.

**Conclusion:**

Older adults with dementia are at an increased risk of poisonings, especially immediately following a dementia diagnosis. Judicious prescribing of high‐risk medications and appropriate management strategies should be prioritised during this time.